# Aplastic Anemia Mimicking Myelofibrosis: A Diagnostic Dilemma

**DOI:** 10.7759/cureus.49445

**Published:** 2023-11-26

**Authors:** Neel Patel, Hamza Mirza, Payal Bai, Vedant Shah, Harsh Patel, Milan Khealani, Geetika Kukreja, Sri J Obulareddy

**Affiliations:** 1 Public Health, Icahn School of Medicine at Mount Sinai, New York, USA; 2 Biology, New Jersey City University, Jersey City, USA; 3 Internal Medicine, Smt. Nathiba Hargovandas Lakhmichand (NHL) Municipal Medical College, Ahmedabad, IND; 4 Clinical Research Management, Rutgers University, Newark, USA; 5 Hospital Medicine, Mayo Clinic Health System, Mankato, USA; 6 Internal Medicine/Hematology-Oncology, Henry Ford Health System, Clinton Township, USA; 7 Hematology Oncology, University of Arkansas for Medical Sciences, Little Rock, USA

**Keywords:** myeloproliferative disorders, myelofibrosis, aplastic anemia, hematological disorder, case report, hematology, blood disorders, bone marrow cancer, jak2 mutation

## Abstract

Hematological disorders pose a diagnostic challenge due to overlapping clinical features, as demonstrated by the difficulty in differentiating between aplastic anemia (AA) and primary myelofibrosis (PM). Myeloproliferative disorders, characterized by aberrant proliferation of bone marrow stem cells, present complexities in diagnosis, often requiring a comprehensive evaluation to distinguish between disorders with similar manifestations. The distinctions between myelofibrosis and AA lie not only in clinical presentations but also in genetic and molecular markers, necessitating a nuanced diagnostic approach.

We present a case of a 37-year-old male initially diagnosed with myelofibrosis based on a history of pancytopenia, warm submandibular and submental swelling, and negative BCR-ABL and JAK2 mutations. Further examination revealed empty fragmented cells, hypoplastic bone marrow, and suppressed erythropoiesis and myelopoiesis. Subsequent core biopsy showed increased megakaryocytes, prompting a revised diagnosis of AA. This case underscores the importance of a meticulous diagnostic journey, incorporating physical examination, genetic testing, and advanced imaging to unravel the complexities of hematological disorders.

The intricacies of this case prompt a reevaluation of diagnostic paradigms, highlighting the limitations of relying solely on specific mutations for diagnosis. The absence of BCR-ABL and JAK2 mutations in AA raises questions about its genetic landscape, necessitating further exploration. Immunological considerations, given the immune-mediated nature of AA, provide a foundation for future research into immune dysregulation and potential therapeutic interventions. The clinical management challenges posed by AA underscore the need for personalized treatment strategies, guided by a deeper understanding of its underlying pathophysiology. Advanced imaging techniques, in conjunction with traditional diagnostic methods, emerge as crucial tools for enhancing diagnostic accuracy in hematological disorders. This case serves as a paradigm for ongoing medical education, multidisciplinary collaboration, and innovative approaches in the evolving landscape of hematology, emphasizing the imperative for continuous refinement in diagnostic strategies and patient care.

## Introduction

Myeloproliferative disorders are a group of disorders that result in an immoderate amount of blood cells. This results in increased production within one or more blood-type cells due to a primary defect that lies within multipotent hematopoietic stem cells which are found in the bone marrow [1]. There are many types of myeloproliferative disorders, but the four most common are polycythemia vera, essential thrombocythemia, primary myelofibrosis (PM), and chronic myelogenous leukemia. PM is characterized by stem cells that continually divide, giving rise to new abnormal cells that interfere with the production of healthy blood cells and leave RBCs that are damaged and immature [2-4].

Myelofibrosis, which is a subset of myeloproliferative disorders, is distinguished by the abnormal accumulation of collagen fibers and fibrous connective tissue in the bone marrow. This process gradually replaces healthy hematopoietic cells with fibrosis. Fatigue, anemia, hepatosplenomegaly, and leukoerythroblastosis are some signs and symptoms that are prevalent in MF patients. The pathogenesis of MF is complex and not yet fully understood, although several genetic mutations, including JAK2, CALR, and MPL, have been identified as contributing factors [5,6]. Generally, there are very limited treatment options for MF. The only curative therapy available is stem cell transplantation. Yet, most patients find it challenging to go for this intensive treatment, as it carries the risk of fatality for some individuals midway through the treatment process. Current treatment including JAK2 inhibitors showed a minimal effect on alleviating the symptoms or changing the prognosis.

Aplastic anemia (AA), on the other hand, presents a distinct hematological challenge, marked by the failure of bone marrow to produce sufficient blood cells, resulting in pancytopenia. Unlike myeloproliferative disorders, AA can be congenital or acquired, with various causative factors such as chemotherapy, radiation, drugs, viral infections, and autoimmune disorders [7,8]. Clinically, AA presents with a range of symptoms, including fatigue, weakness, headaches due to anemia, petechiae, nosebleeds, gum bleeding resulting from severe thrombocytopenia, and susceptibility to infections due to low WBC counts and neutropenia [7]. Diagnosis relies on clinical signs, peripheral blood counts, and bone marrow biopsy results.

In this report, we present a distinctive case of a middle-aged man initially diagnosed with myelofibrosis, who complained of high-grade fever, submandibular, and submental edema. Subsequent in-depth analysis, including genetic testing and bone marrow examination, uncovered AA. This case emphasizes the critical need for meticulous evaluation of hematological disorders to ensure accurate diagnoses and tailored treatment approaches.

## Case presentation

A 37-year-old male patient presented with a one-week history of high-grade fever, submandibular, and submental swelling associated with chills and sweating. The patient had been in his usual state of health seven days prior to the presentation. On physical examination, the submandibular and submental swellings were warm to the touch but non-tender.

The patient was hospitalized for pancytopenia multiple times recently and had received multiple blood transfusions. An initial bone marrow biopsy suggested myelofibrosis. However, further genetic testing yielded negative results for the BCR-ABL mutation and the JAK2 mutation, and chromosomal analysis revealed a normal male karyotype (46XY). Hemoglobin level was 7.3 gram/deciliter (g/dl), urea 69 milligram/deciliter (mg/dL), creatinine 1.3 mg/dL, and electrolytes were within normal limits. Liver function tests were within the normal range. Prothrombin time and international normalized ratio were 13.3 and 1.35 seconds, respectively.

**Figure 1:**
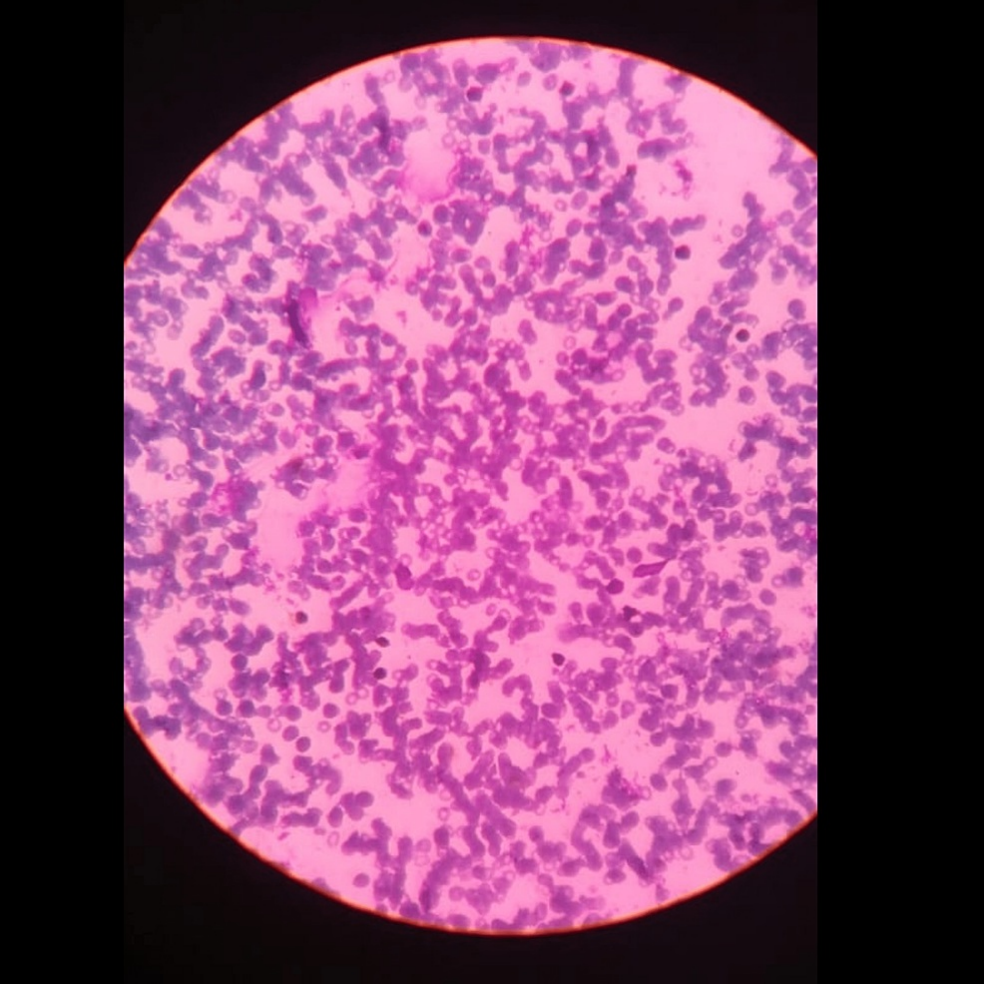
Peripheral smear slide Peripheral smear showing predominantly hypochromic, microcytic RBCs, moderate anisopoikilocytosis, and occasional mature neutrophil. Few fragmented RBCs are also seen. Platelets are not seen. No abnormal or premature cells or parasite is seen.

A bone marrow aspirate from the iliac crest obtained 15 days later revealed empty fragmented cells followed by hypocellular trails. Erythropoiesis and myelopoiesis were suppressed, with a predominance of maturation cell forms. Megakaryocytes, non-erythroid blast content, and hemoparasites were absent. Iron staining demonstrated sparse iron particles in reticular cells (Figure 1). The differential cell counts in the bone marrow aspirate showed myeloid precursor cells at 4%, erythroid precursor cells at 2%, and lymphoid precursor cells at 94% (Table 1). This comprehensive evaluation led to a revised diagnosis of AA.

**Table 1:** Final laboratory results WBC: white blood cell, RBC: red blood cell, PCV: packed cell volume, MCV: mean corpuscular volume, MCH: mean corpuscular hemoglobin, MCHC: mean corpuscular hemoglobin concentration

Test	Results	Normal range
WBC count	300	4,000-11,000/mm^3
RBC count	4.15	4-6 million/mm^3
Hemoglobin	10.7	M14-18 F12-16g/mg/dl
PCV	336	M40-45 F35-40%
MCV	81.0	76-96 fl
MCH	25.7	28-32 pg
MCHC	317	32-36 g/mg/dl
Platelet count	8,000	150,000-400,000 per cmm
Retic count	0.0	0.2-2%
Differential count		
Neutrophils	18	40-75%
Lymphocytes	82	20-45%
Eosinophil	00	01-06%
Monocytes	00	02-10%
Basophils	00	00-01%
RBCs show anisocytosis normochromic, the WBCs show normal morphology, the platelets are inadequate on the smear, and no malarial parasite was seen.		

After the diagnosis of AA, the patient was treated with blood transfusions, supportive care, and close monitoring for any further complications. This case report concludes by highlighting the difficulties in identifying myelofibrosis without the JAK2 mutation present. Although the patient's fever, submandibular swelling, and submental swelling were originally thought to be symptoms of myelofibrosis, AA was eventually determined to be the cause of the patient's symptoms.

Additional investigations, including a core biopsy, revealed increased megakaryocytes and increased cellularity for age in the cellular area. A peripheral blood smear from one month prior showed mild anemia with normocytic, normochromic RBCs, polychromasia, and a reticulocyte count of 1.39%. The WBC differential count showed neutrophils at 55%, lymphocytes at 43%, and monocytes at 2%, with moderately reduced platelets. Importantly, a subsequent bone marrow aspirate showed a dry tap, further supporting the diagnosis of AA. The patient was managed with blood transfusions, supportive care, and close monitoring for any potential complications associated with AA.

## Discussion

Our case highlights the difficulty in differentiating AA from a prior diagnosis of PM due to similar findings. Myeloproliferative disorders are a group of hyperproliferative disorders resulting from multiple alterations in bone marrow stem cells. These conditions manifest as increased peripheral blood cells, bone marrow hyperplasia- excessive proliferation and differentiation of hematopoietic stem cells, and variations in the production rate of RBCs, platelets, or specific WBCs, accompanied by clinical characteristics [2].

Myelofibrosis is a condition characterized by the proliferation of stem cells derived from a single clone, often accompanied by somatic mutations. The mutations can be classified into driver mutations (JAK2, CALR, or MPL) and subclonal mutations along with fibrosis observed in bone marrow biopsy [3]. Myelofibrosis has an incidence of 0.58 per 100,000 individuals and a prevalence of 6 per 100,000 individuals because of its chronicity [4]. The median age of diagnosis for this disorder is 67 years with no variability in sex [4]. AA is an uncommon, potentially life-threatening blood disorder characterized by a reduction in peripheral blood cell counts and bone marrow cell counts affecting all three lineages [9]. AA is diagnosed by bone marrow biopsy, which is made by ruling out other potential causes of bone marrow failure, as there are various possible causes. A proper diagnostic evaluation must be done carefully to examine and exclude alternative causes. In the early stages of AA, patients commonly present fatigue, weakness, headaches (due to anemia), petechiae, nosebleeds, gum bleeding (due to severe thrombocytopenia), and fever and infections (due to low WBC counts and neutropenia) [7].

Both types of bone marrow conditions are brought on by modifications to the stem cells. Moreover, the diagnoses of both are similar in such a way that a bone marrow biopsy is needed. A study by Schoettler et al. discussed the primary cause of AA stems from the destruction of hematopoietic stem and progenitor cells (HSPCs) resulting from a combination of inherent genetic defects within HSPCs themselves and an immune response triggered by a viral infection that is abnormally activated [10].

Our case initially leaned toward myelofibrosis based on the clinical findings and bone marrow biopsy results, supported by negative BCR-ARL and JAK2 mutations, indicating normal cell growth and division (proliferation). However, the subsequent presentation of submandibular and submental swelling, fever, chills, and constitutional symptoms prompted further analysis with iliac crest aspirate and core biopsy. This revealed empty fragmented cells, hypoplastic bone marrow (20% cellularity), fatty bone marrow, a low reticulocyte count, and suppressed erythropoiesis and myelopoiesis at 2% and 4%, respectively. Despite the absence of initially expected findings such as megakaryocytic cells, non-erythroid blast cells, and hemoparasites, the core biopsy ultimately showed increased megakaryocytes, supporting the revised diagnosis of AA. The exact cause of AA, whether constitutional or genetic, immune-mediated destruction of stem cells, or iatrogenic, remained elusive.

This case report concludes by emphasizing the complexities of identifying myelofibrosis without the presence of the JAK2 mutation. The initial impression of myelofibrosis based on symptoms such as fever, submandibular swelling, and submental swelling eventually led to the discovery of AA as the underlying cause, underscoring the importance of continued vigilance in the face of evolving clinical presentations.

Immunological considerations come to the forefront, given the immune-mediated nature of AA. The absence of hemoparasites and non-erythroid blast cells in the bone marrow aspirate, coupled with the subsequent dry tap, suggests a complex interplay of immune mechanisms. Future research could delve into the immunological aspects of AA, exploring immune dysregulation and potential therapeutic interventions targeting the immune system.

The clinical management of the patient involved blood transfusions, supportive care, and close monitoring for complications. The absence of a clear etiological factor raises questions about the optimal treatment approach for AA. The evolving landscape of hematopoietic stem cell transplantation, immunosuppressive therapies, and novel agents targeting specific pathways may offer promising avenues for the management of AA. The challenges in identifying causative factors underscore the need for personalized treatment strategies, emphasizing the importance of a deeper understanding of the underlying pathophysiology.

The diagnostic journey in this case relied heavily on traditional diagnostic modalities such as bone marrow biopsy and aspirate. However, the limitations of these techniques, especially in cases with atypical presentations, highlight the necessity for advanced imaging techniques. Incorporating advanced imaging, such as PET-CT or MRI, may enhance diagnostic accuracy, providing a more comprehensive assessment of bone marrow structure and function.

This case offers valuable insights for clinicians and hematologists, emphasizing the importance of maintaining a vigilant and open-minded approach to diagnostic challenges. The nuances in distinguishing between myelofibrosis and AA underscore the need for ongoing medical education and collaborative efforts to refine diagnostic algorithms. Multidisciplinary collaboration, involving expertise from hematology, immunology, and genetics, can synergize to unravel complex cases.

## Conclusions

This case report underscores the intricate nature of hematological diagnoses and the potential for initial misdiagnoses. What initially appeared as myelofibrosis was ultimately revealed as AA, emphasizing the critical role of comprehensive evaluation and clinical vigilance. This case serves as a valuable reminder for healthcare providers to maintain an open and questioning approach in the face of diagnostic challenges, with the ultimate goal of optimizing patient care and outcomes.
